# Strength Training Volume to Increase Muscle Mass Responsiveness in Older Individuals: Weekly Sets Based Approach

**DOI:** 10.3389/fphys.2021.759677

**Published:** 2021-09-30

**Authors:** Davi Alves de Santana, Alex Castro, Cláudia Regina Cavaglieri

**Affiliations:** ^1^Laboratory of Exercise Physiology, Faculty of Physical Education, University of Campinas, Campinas, Brazil; ^2^Adventist University of Sao Paulo, São Paulo, Brazil; ^3^Nuclear Magnetic Resonance Laboratory, Department of Chemistry, Federal University of São Carlos, São Carlos, Brazil

**Keywords:** resistance training, aging, non-responsiveness, hypertrophy, sets

## Introduction

Currently, strength training (ST) is widely recommended to promote healthy aging. This reflects efforts made over the past three decades through which the role of ST as a sarcopenia countermeasure, preventing physical disability and other poor outcomes, has become evident. However, in recent years, considerable attention has been directed to the phenomenon of ST response heterogeneity. Different adaptive patterns among individuals submitted to the same intervention have increasingly led scholars to conventionally label non-responders or low responders those who do not respond appropriately, either for lack of meaningful improvements or even for worsening parameters. In the latter case, such individuals are specifically classified generally as adverse responders since they present responses in opposite direction to a threshold theoretically or empirically determined (Bouchard et al., 2012; Hecksteden et al., 2018). Although there is still no consensus on the definition of non-responders or low responders to ST, showing that their study is in its infancy, an interesting review by Atkinson et al. (2019) suggests that the categorization of a given response should be rationalized by the researcher after analyzing which threshold is clinically or practically relevant, changing the notion that minimal detectable change is imperative to determine who respond or not to an intervention.

In this scenario, the phenomenon of non- or low-responsiveness indirectly favored the development of a more restrictive and less important perspective regarding the role of ST. However, reaffirming the ST relevance in aging, Churchward-Venne et al. (2015) have claimed that there are no older non-responders. In that study, it has been shown that all older adults submitted to ST improve at least one analyzed parameter (e.g., functional capacity and muscle strength), defended its widespread application. As suggested by Pickering and Kiely (2019), there are likely no individuals totally unresponsive to training. Nevertheless, the reason why some individuals show less expressive results than others in apparently homogeneous samples remains unclear, especially concerning muscle hypertrophy.

It can be noticed in the study conduct by Churchward-Venne et al. (2015) that more than 35 and 30% of the older individuals had a maximum lean mass increase of 0.5 kg after 12 and 24 weeks of ST, respectively. In addition, about 20% had a decrease in lean mass, regardless of protocol duration. Despite the inherent physiological changes of aging, Ahtiainen et al. (2016) demonstrate that the variability of muscle mass response to ST is not affected by age. Although such changes may indeed lead older individuals to a decreased skeletal muscle tissue sensitivity to anabolic stimuli (Yang et al., 2012), it prohibits putting on account of aging the occurrence of inexpressive morphological adaptations in response to training exclusively. Regarding this muscle mass response heterogeneity, while some scholars (Atkinson et al., 2019; Dankel and Loenneke, 2020) recommend cautiousness when claiming its existence, emphasizing that individual differences need to be attested in studies that consider the random error, verified from a matched control group, many others widely recognize it (Hubal et al., 2005; Davidsen et al., 2011; Sparks, 2017; Stec et al., 2017; Camera, 2018; Räntilä et al., 2021). Although more studies need to be conducted to demonstrate the true variability of the response, the body of evidence indicates that such heterogeneity should not be ignored. In this context, it is inevitable to admit that more efforts should be made to clarify the reasons behind the low-responsiveness of some individuals, which, in turn, might assist in planning more effective ST programs.

## Volume: A Key Point?

The proper design of an ST program involves the management of several variables. Among all, most of them are closely related to intensity and volume. By intensity, it is recognized as the load lifted in a given movement, calculated basically by the percentage of the maximum load lifted only once [e.g., percentage of one-repetition maximum (1RM)]. The training volume, in turn, is traditionally determined by multiplying the number of sets, repetitions, and load. It is noted that the amount of load lifted directly influences the number of repetitions performed (Spiering et al., 2008). Because of the close relationship between repetitions and load, the number of sets performed plays an independent role during the ST progression management and training volume measurement.

Currently, some scholars have considered volume the most effective ST variable to promote morphological changes (Figueiredo et al., 2018). Mattocks et al. (2017) demonstrated that subjects submitted to very high-intensity strength training, simulating 1RM test sessions, achieved the same strength gains as those who trained at a higher volume. However, only the group that trained with higher volume presented muscle hypertrophy. From a molecular point of view, higher exercise volume positively affects myofibrillar protein synthesis and anabolic signaling and is critical for the degree of p70S6k and S6 phosphorylation following a resistance exercise bout (Burd et al., 2010a,b; Terzis et al., 2010). Additionally, it appears that the type of contraction is not more important than volume to cause molecular changes after an exercise session (Garma et al., 2007). A meta-analysis on the influence of ST on the lean mass of the older individuals found that more significant results were particularly related to protocols where more total sets were performed during the training session (Peterson et al., 2011).

From this evidence, it would be reasonable to expect greater effectiveness of experimental protocols on morphological as higher training volume is applied. Several studies comparing the performance of a single set with multiple sets have demonstrated the superiority of performing multiple sets in both young and older individuals (Kramer, 1997; Radaelli et al., 2014a,b). However, the meta-analysis conducted by Krieger (2010) minimizes the relevance of the volume. In this review, the authors concluded that multiple sets are, in fact, better than a single set but performing four to six sets is not superior to performing two to three sets. While providing valuable information, this body of evidence partially explains why increasing the training volume for more robust physiological responses is not a unanimous strategy (Souza et al., 2018). By the way, it seems that there is a subtle scientific orientation in establishing a minimum dose of exercise by which older people can benefit from ST to the detriment of the search for an optimal dose (Fisher et al., 2017). This is understandable because less time spent on training sessions may increase adherence to the intervention.

These conflicting perspectives raise some unavoidable and related issues. First, does an inadequate training volume explain the unresponsiveness of some older individuals? Consequently, is it possible to establish an optimal volume to maximize hypertrophy in this population? To the best of our knowledge, only one study attempted to answer directly whether training volume is important for the variability of response to ST in older people, despite the large body of studies on this topic (Nunes et al., 2021b). Nunes et al. (2021b) verified that the non-responsiveness condition was not changed after the training volume increase. However, one should be cautious to make conclusions based on this evidence, since it is an analysis of retrospective data in which randomization was not specifically performed to provide a comparative analysis of training volume. On the other hand, Scarpelli et al. (2020) showed that a suboptimal training volume could hamper muscle hypertrophy responses in trained young individuals. This issue is yet to be impulsed by the interesting protocol designed by Montero and Lundby (2017), in which it was observed that all individuals classified as non-responders after 6 weeks of aerobic training responded positively after the additional 2 weeks with higher training volume. Although the intervention of this study has been aerobic training, one can speculate that non-responders to ST also may achieve better adaptations naturally by increasing the training volume.

## Weekly Sets Per Muscle Group

Despite current recommendations on ST for older people (Fragala et al., 2019), which suggests prescribing two to three sets per exercise based on the previously discussed evidence, the optimal training volume to increase muscle mass responsiveness in older individuals is still a scientific challenge. It is proposed here that the findings observed in the literature may be interpreted based, specifically, on the number of weekly sets per muscle group to allow a more comprehensive analysis on this issue, since from this perspective the number of exercise and weekly frequency are already taken into account, avoiding misleading conclusions. It is worth mentioning that these factors usually differ among studies comparing single and multiple sets, which may partially be responsible for obtaining different results. Thus, this approach would contribute to a greater connection among the findings, uniformity in experimental design conception, and practicality in the training prescription, emphasizing that few studies analyzed volume from this perspective.

In this regard, an interesting meta-analysis elaborated by Schoenfeld et al. (2017) provides one of the main clues on this matter. They investigated the dose–response relationship between weekly ST volume and muscle mass gains analyzing studies that compared protocols with low and high training volumes. It was noted that ST tends to promote better results with higher doses, further concluding that 10 weekly sets per muscle group or more may be necessary to maximize muscle hypertrophy. Even though this review was not limited to the older individuals, this is consistent with the review proposed by Peterson et al. (2011), although they did not specifically address the number of weekly sets per muscle group. It can be inferred that one of the main strengths of the analysis by Schoenfeld et al. (2017) was to indicate that there may be a training volume threshold to be reached. Importantly, this finding demands a more critical appraisal of current literature since some clinical trials claiming to apply a high training volume may be less than the supposedly required.

While a reduced number of weekly sets may be insufficient to promote more significant hypertrophy, excessive sets may produce prolonged muscle damage and consequently, decrease adaptive response in older people chronically (Hamada et al., 2005; Ferri et al., 2006; Fell and Williams, 2008; Sorensen et al., 2018). Yet, the dose–response of volume on muscle damage in older individuals has to be determined. In this regard, it was observed that 15 weekly sets per muscle group causes more ultrastructure muscle damage in older than young women after a 9-week intervention (Roth et al., 2000) but not in older men when compared to young (Roth et al., 1999). Although one may argue that the high intensity proposed in these studies may have influenced the results, it has been shown that high and low-intensity training with equal volume produces the same level of muscle damage in young people (Paschalis et al., 2005). It is reasonable to suppose that some older people, especially women, do not respond adequately to training due to overtraining, which in turn makes the previous results (Krieger, 2010) understandable since increasing training volume (2 to 3 vs. 4 to 6 sets a week) does not necessarily improve response. Therefore, it appears that there is an upper threshold for ST volume in older individuals still have to be confirmed. This observation may be particularly relevant in designing training programs for frail individuals and others with chronic diseases since higher training doses could be more harmful.

In this scenario, training weekly frequency should not be neglected. Dankel et al. (2017) have hypothesized that higher training frequencies may induce a higher hypertrophic response by promoting optimal successive increase in protein synthesis rate. Based on the evidence that there is a plateau in protein synthesis response to increased training volume (Kumar et al., 2012; Martín-Hernández et al., 2013), there would be no advantage in performing a high training volume in a single session. However, it is not possible to make inferences in this regard from the current evidence. A meta-analysis conducted by Schoenfeld et al. (2019) concluded that the ST frequency does not meaningfully impact muscle hypertrophy when the volume is equated. Concerning older individuals, recently Pina et al. (2019) found no difference in lean mass response between individuals who trained two or three times a week after a 12-week sets-equated ST. On the other hand, Zaroni et al. (2019) found that high-frequency training (five vs. one time per week) may confer a superior hypertrophic response in young individuals. Regarding the comparison of protocols, Nunes et al. (2021a) argued that weekly set-equated do not necessarily mean volume-equated when there are marked differences in the weekly frequency (≥3) since a reduction in the number of repetitions performed in groups with low frequency is observed due to fatigue experienced during the sessions. Nevertheless, some studies that compared protocols with markedly different frequencies with weekly sets-equated did not find differences in the increase in muscle hypertrophy (Gomes et al., 2019; Saric et al., 2019).

We recognize that further research needs to be carried with the older population in this sense, as previously suggested (Dankel et al., 2017). Meanwhile, the number of weekly sets needed to enhance muscle hypertrophy in low-responsive older individuals could be achieved by following the ST current position, which recommends a frequency of two to three times per week, comprising one to two exercise for each muscle group per session (Fragala et al., 2019), depending, however, on sets number per exercise, in which it must be individually adjusted during the exercise program.

## What Is Next?

To provide more specific recommendations regarding the proper range of weekly sets per muscle group to increase responsiveness to ST in older people, it is first necessary that the scientific community does not ignore the heterogeneity of the exercise response, reflected, often, by neglecting individual responses in clinical trials. This simple but significant perspective may contribute greatly to the characterization of the non-responsiveness phenomenon and its prevalence, especially with respect to muscle mass. In particular, there should be an effort to define the minimal clinically important difference regarding muscle hypertrophy in older individuals to support responsiveness categorization, since such difference hardly coincides with the minimum detectable change (Atkinson et al., 2019). Thus, clinical trials assessing relevant clinical outcomes together with hypertrophy response at different times of intervention are required. Second, as a few reviews addressing the dose–response effects of training volume in the older individuals were inconclusive (Steib et al., 2010; Nicola and Catherine, 2011; Silva et al., 2014; Borde et al., 2015) or did not assess morphological adaptations (Rhea et al., 2003), systematic reviews and meta-analysis on the dose–response relationship between weekly sets per muscle group, rather than exercise general sets or weekly frequency, and morphological adaptations in this population are encouraged. It is worth mentioning, none of the reviews cited above considered or measured individual responses. Third, dose–response should be investigated through within-subject experimental protocols and long-term interventions. On the latter, if, on the one hand, the duration of the intervention has been considered one of the most relevant ST variables for elderly individuals (Nicola and Catherine, 2011; Radaelli et al., 2014b), on the other hand, prolonged interventions could minimize the influence of the training volume on the hypertrophic response (Da Silva et al., 2018; Teodoro et al., 2019). In future investigations, it is imperative to separate the true interindividual response variance from other sources of variance, since the individual observed changes are the sum of the change caused by the training program, plus the change that would have occurred in the absence of intervention (in the control group), plus measurement error and day-to-day biological variability (Ross et al., 2019). We also emphasize controlling confounders such as concerning the dietary pattern including energy balance and protein intake, given their potential role on muscle mass maintenance (Houston et al., 2008; Hanach et al., 2019; Landi et al., 2019). In addition, possible differences between lower and upper-body muscle group's responses to training should be considered, as there is evidence that high-volume training seems to be more efficient for lower-body muscles (Radaelli et al., 2014b). Lastly, alternative assessments are necessary to understand the variability of the response and assist in planning new studies. In this sense, omic sciences (e.g., genomics, proteomics, and metabolomics) emerge as the next frontier to be explored in this field of knowledge (Tanaka et al., 2016; Picca et al., 2019). From them, diverse dimensions of the organism (e.g., metabolic and genetic) are analyzed simultaneously and, when associated with training responses, can create network maps that help to explain the complexity of morphological adaptations to ST.

## Considerations

Strength training is an important strategy against poor outcomes during aging. In order to enable that older individuals plenty achieve positive adaptations, training variables need to be carefully manipulated. In this context, although it is recognized that the training volume is fundamental for muscle hypertrophy, there is no consensus on the magnitude of its influence. Consequently, the contribution level of the volume training for increasing responsiveness to ST in older individuals is lacking. It is noticeable that establishing such a volume is a challenge to be addressed. While future studies are expected to clarify this issue, it can be hypothesized, based on current evidence, that there should be a more specific range of training volume that older people can respond more adequately. We suggested that such a range could be based on the number of sets per muscle group, comprising at least 10 sets per week. Evidently, this does not mean that older individuals do not respond to other training volumes (e.g., <10), but only that some non-responders or low-responders may have better morphological adaptations in a specific range. In practical terms, after an initial adaptation period, the training volume can be allocated in this range and adjusted according to individual responses to achieve better results. To reach this number of sets, one must carefully select exercises for each muscle group and weekly frequency. In addition, reporting ST volume based on weekly sets per group muscle in future studies may facilitate literature analysis as it already includes the aforementioned variables.

## Author Contributions

DS contributed to the conception of the study and wrote the first draft of the manuscript. AC and CC revised the original manuscript critically for important intellectual content. All authors read and approved the final manuscript.

## Funding

AC was supported by the São Paulo Research Foundation (FAPESP, Grant No. 2020/13939-7).

## Conflict of Interest

The authors declare that the research was conducted in the absence of any commercial or financial relationships that could be construed as a potential conflict of interest.

## Publisher's Note

All claims expressed in this article are solely those of the authors and do not necessarily represent those of their affiliated organizations, or those of the publisher, the editors and the reviewers. Any product that may be evaluated in this article, or claim that may be made by its manufacturer, is not guaranteed or endorsed by the publisher.
